# Therapeutic Notes

**Published:** 1896-09-19

**Authors:** 


### THERAPEUTIC NOTES.

### LYSOL AS AN ANTISEPTIC.

Lysol was introduced in Germany as an antiseptic some seven years ago, and has in that country enjoyed an excellent reputation as an antimycotic and disinfectant. It is a dark-coloured alkaline liquid, obtained by the saponification of cresols and containing the higher homologues of carbolic acid; it mixes in all proportions with water, and is generally used in about 1 per cent. solution. It is highly poisonous, though reputed to be less so than carbolic acid. A considerable number of cases of poisoning with this German speciality have been recorded, and a few have ended fatally. The symptoms appear to be much the same as those of carbolic acid poisoning, and the treatment is practically similar. Its chief use has so far been found in obstetric practice,[1] being of considerable value as a douch when fœted discharges are present; it should not be used in stronger solution than 1 per cent. for this purpose, and even then its application is attended with a certain amount of smarting. In surgical practice it may be used also in 1 per cent. strength and 2 per cent. for the instruments; it has the advantage of affording a good lather, and thus may be used as a substitute for soap in cleaning the hands or greasy instruments; it is said to cause no irritation of the skin in ordinary strength, although in more concentrated solution, it causes acute inflammation and eccymosis.[2] It has been recommended as an internal antiseptic,[3] and is stated by Maas to be well tolerated in doses of 0 05—0 5 grams. and may be given in the form of pill, capsule, or aqueous solution. It can be given in combination with alkalis, but not with acids, and as a rectal injection in cases of dysentery, it is said to be particularly efficacious by Rudneff.[4] One pint of a one per cent. solution should for this purpose be injected three times daily, but since carbolic[5] acid, when used in the same manner has caused death, it should be used with caution. The poisonous dose is stated to be 0 08 grams. per kilogram body weight. When poisoning occurs through the stomach, the latter organ should be washed[6] out, and five grams of suspended magnesia pumped into the viscus.[7]

[1] Isnealron, S. Petersburg, Med. Woch. No. 47, 1893. [2] Dr. Saalfeld, Therap. Monats, 1896, fasc ent v., p. 201. [3] Deuch. Arch. für Klin. Med., 1894, Vol. 52. [3] Meditz Obozreine. No. 20, 1893, p. 747. [4] Friedeburg, Cent. blat. für inna Med., March 3, 1894. [5] Ærzl, p. 35. [7] Central blat. für inn. Med. iii., 1894.

